# Event-related brain potential markers of visual and auditory perception: A useful tool for brain computer interface systems

**DOI:** 10.3389/fnbeh.2022.1025870

**Published:** 2022-11-29

**Authors:** Alice Mado Proverbio, Marta Tacchini, Kaijun Jiang

**Affiliations:** ^1^Laboratory of Cognitive Electrophysiology, Department of Psychology, University of Milano-Bicocca, Milan, Italy; ^2^Department of Psychology, University of Jyväskylä, Jyväskylä, Finland

**Keywords:** EEG/ERP, mind reading, brain computer interface (BCI), semantic categorization, perception

## Abstract

**Objective:**

A majority of BCI systems, enabling communication with patients with locked-in syndrome, are based on electroencephalogram (EEG) frequency analysis (e.g., linked to motor imagery) or P300 detection. Only recently, the use of event-related brain potentials (ERPs) has received much attention, especially for face or music recognition, but neuro-engineering research into this new approach has not been carried out yet. The aim of this study was to provide a variety of reliable ERP markers of visual and auditory perception for the development of new and more complex mind-reading systems for reconstructing the mental content from brain activity.

**Methods:**

A total of 30 participants were shown 280 color pictures (adult, infant, and animal faces; human bodies; written words; checkerboards; and objects) and 120 auditory files (speech, music, and affective vocalizations). This paradigm did not involve target selection to avoid artifactual waves linked to decision-making and response preparation (e.g., P300 and motor potentials), masking the neural signature of semantic representation. Overall, 12,000 ERP waveforms × 126 electrode channels (1 million 512,000 ERP waveforms) were processed and artifact-rejected.

**Results:**

Clear and distinct category-dependent markers of perceptual and cognitive processing were identified through statistical analyses, some of which were novel to the literature. Results are discussed from the view of current knowledge of ERP functional properties and with respect to machine learning classification methods previously applied to similar data.

**Conclusion:**

The data showed a high level of accuracy (*p* ≤ 0.01) in the discriminating the perceptual categories eliciting the various electrical potentials by statistical analyses. Therefore, the ERP markers identified in this study could be significant tools for optimizing BCI systems [pattern recognition or artificial intelligence (AI) algorithms] applied to EEG/ERP signals.

## Graphical Abstract

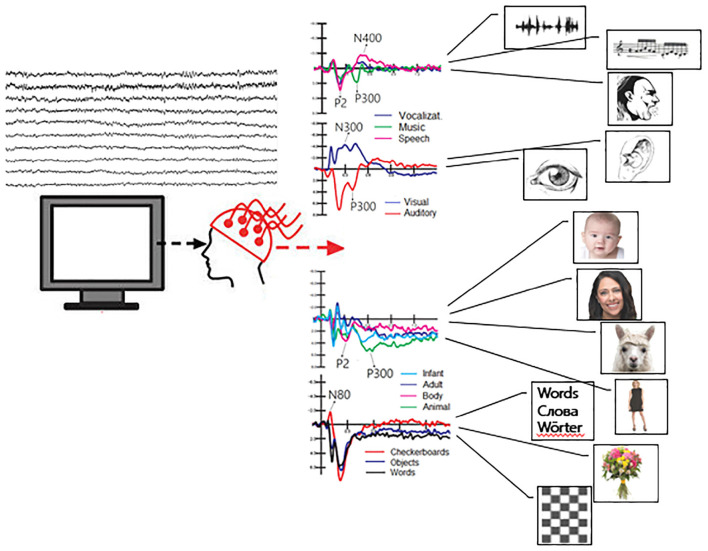

## Introduction

A BCI system is a device that can extract brain activity and process brain signals to enable computerized devices to accomplish specific purposes, such as communicating or controlling prostheses. The more commonly used systems involve motor imagery (e.g., Hétu et al., 2013; Kober et al., 2019; Su et al., 2020; Jin et al., 2021; Milanés-Hermosilla et al., 2021; Mattioli et al., 2022), communication (Blankertz et al., 2011; Jahangiri et al., 2019; Panachakel and G, 2021), face recognition (Zhang et al., 2012; Cai et al., 2013; Kaufmann et al., 2013), or P300 detection (Pires et al., 2011; Azinfar et al., 2013; Guy et al., 2018; Shan et al., 2018; Mussabayeva et al., 2021; Rathi et al., 2021; Leoni et al., 2022). Only few studies have used simultaneously BCI systems for the recognition of multiple ERP signals reflecting distinct types of mental contents, such as music (Zhang et al., 2012), faces (Cai et al., 2013; Li et al., 2020), or visual objects (Pohlmeyer et al., 2011; Wang et al., 2012). Indeed, ERP potentials, since their discovery about 40 years ago (Ritter et al., 1982), have proven to be a quite reliable marker of category-specific visual and auditory processing (see also Zani and Proverbio, 2002; Helfrich and Knight, 2019).

Quite recently, an interesting study applied machine learning algorithms for blindly categorizing ERP signals as a function of the type of stimulation (Leoni et al., 2021). The dataset consisted of grand average ERP waveforms related to 14 stimulus categories of stimulation (the same stimuli used in the present studies together with video stimuli), recorded from 21 subjects. A total of two classification models were designed, each based on machine learning algorithms. In detail, one model was based on boosted trees, and the other model was based on feed-forward neural networks. Considering accuracy performances, both the models exceeded the minimum threshold to guarantee meaningful communication (∼70%). The accuracy performance for discriminating checkerboard images from other images representing tools and objects was 96.8%. Considering the temporal cut approach, the best performance was obtained in discriminating audio stimuli from visual stimuli (99.4% for both boosted trees and 100% for feed-forward neural networks). The worst performance was obtained in distinguishing images representing living (e.g., faces and bodies) from non-living entities (e.g., objects and words), where boosted trees achieved an accuracy of 86.9% and feed-forward neural networks achieved an accuracy of 99.6%. Since the classification of neuroscientific knowledge of the functional significance, timing, and scalp localization of ERP components was blindly performed, the results seem quite interesting and very promising. However, the binding with neuroscientific knowledge may supposedly increase the level of accuracy for setting-specific constraints. Statistical analyses applied to amplitude values of ERP components (e.g., Picton et al., 2000; Zani and Proverbio, 2002; Dien, 2017), in determining if the variance of recorded electrical voltages can be reliably “explained” by the semantic category of stimulation (e.g., faces vs. objects), may indeed reach a higher level of significance (e.g., 99.9 or 99.99%). Therefore, in addition to non-expert classification approaches, it can be very useful to use statistics to categorize ERP signals according to the type of stimulation by applying expert knowledge of the spatiotemporal coordinates of electrical potentials. Each peak in the scalp area (e.g., the P300) reflects the inner activity of simultaneous neural sources reaching their maximum degree of activation at a given latency and area, while spreading nearby for volume conduction with smaller voltage amplitudes (see Zani and Proverbio, 2002 for further discussion). Therefore, measuring the point at which the peak reaches its maximum amplitude (Picton et al., 2000) more closely reflects the brain activity related to the specific processing stage (e.g., decision-making and feature analysis). The purpose of this study was to identify a set of reliable psychophysiological markers of perceptual processes by using the EEG/ERP recording technique.

Various neurophysiological studies have investigated the neural processes associated with the encoding of different visual and auditory stimuli. These studies have highlighted the time course and neural substrates of category-specific processing. Neuroimaging studies, on the other hand, have offered consolidated knowledge of the existence of specific brain areas reflecting category-specific neural processing. For example, the fusiform face area (FFA, Kanwisher et al., 1997; Haxby et al., 2000) is thought to be sensitive to configural face information. The extra-striate body area (EBA) is particularly sensitive to body perception (Downing et al., 2001), while the parahippocampal place area (PPA) strongly responds to places and houses.

Similarly, the ERP literature has provided reliable evidence of bioelectric markers of category-specific processing. For example, there is long-standing evidence that human faces evoke a negative potential (N170), peaking after about 170 ms from the stimulus onset over the posterior occipitotemporal area, mostly right-sided in male individuals (Proverbio, 2021). N170 is smaller for non-face than face stimuli (Bentin et al., 1996; Liu et al., 2000; Proverbio et al., 2010; Rossion, 2014; Gao et al., 2019), and larger and delayed for inverted faces as compared with that for normal upright faces (Rossion and Gauthier, 2002). N170 is also larger for infant faces (baby schema) than for adult faces (Proverbio et al., 2011b,2020b; Proverbio and De Gabriele, 2019). According to the literature, the sight of infant faces would trigger a reward response in the orbitofrontal cortex of the adult brain. This nigrostriatal activation would be correlated with the psychological sensation of cuteness and tenderness (Bartles and Zeki, 2004; Kringelbach et al., 2008) and with the development of an anterior N2 response of ERPs, enhanced by the perception of infant vs. adult faces (Proverbio et al., 2011b; Proverbio and De Gabriele, 2019). Rousselet et al. (2004) found that N170 elicited by animal faces was similar in amplitude to that elicited by human faces, but with a delayed peak latency.

Proverbio et al. (2007), using a perceptual categorization task involving images of animals and objects, found that at early processing stages (120–180 ms), the right occipitotemporal cortex was more activated in response to the images of animals than objects as indexed by a posterior N1 response, while frontal/central N1 (130–160 ms) showed the opposite pattern. Table 1 summarizes the functional properties of N170 and other face-specific brain waves. Human bodies are processed in a specific area called EBA of the brain. This region is located in the lateral occipitotemporal cortex and selectively responds to visual images of human bodies and body parts, with the exception of faces (Downing et al., 2001). Another area, called fusiform body area (FBA), which is located on the middle fusiform gyrus of the right temporal lobe, shows selective activity to the visual appearance of the whole body. Precisely, the EBA seems to be more involved in the representation of individual body parts, while the FBA appears to preferentially represent larger portions of the body (Taylor et al., 2007). Various studies indicate that human bodies or body parts produce a response similar to N170 for faces. Moreover, for both bodies and faces, this response is enhanced and delayed by image inversion, indexing configural processing, and is reduced by image distortion (Peelen and Downing, 2007). Table 2 summarizes the functional properties of N170/N190 and other body-specific brain waves. Thierry et al. (2006) found a strong response to bodies that peaked at 190 ms and was generalized to stick figures and silhouettes, but not to scrambled versions of these figures.

**TABLE 1:** Functional properties of N170 and other face-specific responses of visual ERPs to infant, adult, and animal faces, according to psychophysiological literature (Bentin et al., 1996; Kanwisher et al., 1997; Haxby et al., 2000; Rossion and Gauthier, 2002; Rousselet et al., 2004; Proverbio et al., 2006, 2007, 2020b; Sadeh and Yovel, 2010; Noll et al., 2012; Rossion, 2014; Wiese et al., 2014; Jacques et al., 2019; Proverbio, 2021).

Faces				

Children faces				

Peak	Latency (in ms)	Scalp area	Electrodes	Functional properties
N170	150–190	Occipito- temporal cortex; generated within the FFA and the inferior occipital gyrus (IOG)	PPO9h, PPO10h, P7, P8	Larger to infant than adult faces; modulated by children’s facial expressions (larger to negative emotions) and greater in women than men. Its amplitude is modulated by depression symptoms in mothers. N170 is not modulated by baby ethnicity
N2	200–350	Orbito-frontal areas; reflecting the reward response to baby-schema	Fpz, Cz, AFF1, AFF2	Larger for infant than adult and animal faces

**Adult faces**				

N170	150–190	FFA; Right posterior temporal region in males; Occipitotemporal cortex	PPO9h, PPO10h, P7, P8	Larger over the right hemisphere in men, more bilateral in women. N170 peaks earlier for upright than inverted faces. It is larger to inverted than upright faces. It is larger to other- than own-race faces (ORE effect) and to face than non-face stimuli

**Animal faces**				

N170	150–190	Right occipitotemporal cortex; FFA	PPO9h, PPO10h, P7, P8	Similar amplitude compared to human faces, but with delayed peak latency. N1 larger for animals’ faces than for other objects in other studies
N2	200–350	Posterior temporal and posterior occipital sites	PPO9h, PPO10h, P7, P8	Shorter latency at posterior temporal and posterior occipital sites in response to animals than objects
P300	300–350	Centroparietal sites	CPz, Pz	Larger amplitude to animals’ faces than objects

The statistical significances for category discrimination were mostly at a *p*-value < 0.01 or higher.

**TABLE 2:** Functional properties of several body-specific ERP components according to the literature (Downing et al., 2001; Thierry et al., 2006; Peelen and Downing, 2007; Taylor et al., 2007).

Bodies				
Peak	Latency (in ms)	Scalp area	Electrodes	Functional properties
N170	150–190	Occipitotemporal cortex	PPO9h, PPO10h, P7, P8	Larger amplitude and longer latency for inverted than for upright bodies
N190	130–230	Lateral temporo-occipital regions. Generated within Extra-striate Body Area (EBA) and Fusiform Body Area (FBA)	PO7, P7, PO9, O1, O2, P8, PO10, PO8	Larger amplitude for human bodies than for other categories. Larger amplitude and longer latency for inverted than for upright bodies
P2	300–350	Centroparietal sites	Pz, Cpz	Larger amplitude for bodies than for animals, adult and infant faces

According to solid neuroimaging literature, orthographic stimuli such as words and letter strings would be preferentially processed by a specialized area of the left infero-temporal cortex named the *visual word form area* (VWFA) (McCandliss et al., 2004; Lu et al., 2021). Its electromagnetic manifestation would be a negative and left-sided component peaking at about 170 ms over the occipitotemporal scalp area. N170 is larger for words than for letter strings (Simon et al., 2004), and larger for letter strings than for non-orthographic stimuli (Proverbio et al., 2006). MEG studies on adults with dyslexia showed inadequate activation of mN170 during reading (Helenius et al., 1999; Salmelin et al., 2000).

One of the largest and more reliable linguistic components is the centro/parietal N400 usually reflecting a semantic incongruence between a stimulus and its semantic context (as in the sentence: “John is drinking a glass of *orthography*”). According to Kutas and Iragui (1998), N400 is also sensitive to word frequency, orthographic neighborhood size, repetition, semantic/associative priming, and expectancy. The opposite of N400 is the P300 component, which reflects cognitive update, working memory, and comprehension. Table 3 summarizes the functional properties of orthographic N170 and other linguistic brain waves.

**TABLE 3:** Functional properties of orthographic N170 and major linguistic components according to the current ERP literature (Nobre and McCarthy, 1995; Kutas and Iragui, 1998; Helenius et al., 1999; Salmelin et al., 2000; Proverbio et al., 2004b,^2006^, ^2008^; Simon et al., 2004; Lau et al., 2008; Kutas and Federmeier, 2009; Lewendon et al., 2020; Brusa et al., 2021).

Written words				
Peak	Latency (in ms)	Scalp area	Electrodes	Functional properties
N170	150–190	Occipitotemporal regions	P7, P8, PPO9h, PPO10h	Larger for orthographic items than for strings of pseudo-letters. Sensitive to lexical properties. Larger over the left hemisphere. Bilateral, smaller in amplitude and insensitive to orthographic properties in surface dyslexia
N2	250–350	Left occipitotemporal region	PPO9h, PPO10h, POO9h, POO10h	Larger to high- than low-frequency words or pseudo-words
Phonologic mismatch negativity	150–350	Frontocentral	F3, F4	Visual or auditory. Reflecting phonologic incongruence and grapheme-phoneme conversion. Sensitive to audio-visual incongruence
N400	300–500	Centroparietal sites at scalp. Presumably generated within the left medial temporal gyrus	CPZ, CP3, CP4	Larger to semantically inappropriate, incongruent, or unexpected words. Sensitive to close probability, familiarity, frequency, semantic relatedness, world knowledge, and prejudices. Usually larger over the right side at scalp

For shortness, we have omitted the description of other grammar-related linguistic components, such as early late anterior negativity, late anterior negativity, lexical processing negativity, P600, and syntactic positive shift (SPS).

Previous neuroimaging studies have provided evidence for the existence of visual areas devoted to object recognition, mostly within centrotemporal areas BA20/21 (e.g., Uecker et al., 1997; Bar et al., 2001). There seems to exist a functional dissociation between manipulable and non-manipulable objects, with an occipitotemporal activation for object processing and additional activation of premotor and motor areas for tool processing (e.g., Grafton et al., 1997; Creem-Regehr and Lee, 2005).

On the electrophysiological domain, there is no clear evidence of category-specific ERP markers for distinct object categories, apart from N1 to N2 visual responses that, in general, reflect object perceptual analysis within the ventral stream. As for the distinction between tools and non-tools, it was found that tools and non-manipulable objects elicited a larger anterior N2 (210–270 ms) response. Proverbio et al. (2011a) found larger AN (210–270 ms) and a centroparietal P300 (550–600 ms) in response to graspable tools than in response to objects, particularly over the left hemisphere. The occipitotemporal cortex was identified as the most significant source of activity for familiar objects, while the left postcentral gyrus and left and right premotor cortices as the most significant sources of activity for graspable tools. Allison et al. (1999) identified an object-specific N2 response to complex and scrambled objects, which suggests its sensitivity to configural object features. Proverbio et al. (2004a) found a larger occipitotemporal N2 response to target objects depicted in their prototypical vs. unrelated color, suggesting that it reflected the activity of a putative *object color knowledge* area (Chao and Martin, 1999). Again, Orlandi and Proverbio (2019) found a midtemporal N2 response sensitive to the orientation of familiar objects (wooden dummies, chairs, Shepherd cubes). Table 4 summarizes some functional properties of the main visual components reflecting a preference for object categories.

**TABLE 4:** ERP responses elicited by different categories object categories (Allison et al., 1999; Kenemans et al., 2000; Proverbio et al., 2011a,^2021^; Orlandi and Proverbio, 2019).

Patterns and objects				
Peak	Latency (in ms)	Scalp area	Electrodes	Functional properties
N40	30–60	Occipitoparietal, mostly reflecting cortico/thalamic potentials	POZ, PZ. PO1, PO2	Sensitive to grating spatial frequency
N80	60–80	Occipitoparietal and mesial occipital, mostly reflecting V1 activity	POZ, PZ, O1, O2, PO1, PO2	Sensitive to grating and checkerboard spatial frequency and orientation
N2	250–350	Anterior and inferior frontal region	AF3, AF4, AF7, AF8	Larger to graspable than non-graspable objects
P300	550–600	Centroparietal sites	Pz, Cpz	Larger to graspable tools than objects

For shortness, we have omitted the description of lateral occipital P1, occipitotemporal N170, and posterior N2 components (e.g., Muñoz et al., 2020) strongly sensitive to object features and attention but which are not clear markers of categorical processing.

With regard to the auditory stimuli used in this study, neuroimaging data have provided striking evidence of some (functional and anatomical) dissociations between brain regions supporting music, speech, or vocalization processing. If the superior temporal lobe, as well as Heschl gyri for simple acoustic features, is involved in the processing of all of the aforementioned stimuli, phonetic processing will involve STG bilaterally, the SMA bilaterally, the right posterior IFG and premotor cortex, and the anterior IPS bilaterally (e.g., Binder et al., 2008). Again, it seems that dorsomedial regions of the temporal lobe would more reliably respond to music, while ventrolateral regions would more reliably respond to speech (Tervaniemi et al., 2006). The posterior auditory cortex would be more sensitive to pitch contour, while the anterior areas would be more sensitive to pitch chroma (Zatorre and Zarate, 2012). Affective vocalization would activate the anterior and posterior middle temporal gyri (MTG), inferior frontal gyrus, insula, amygdala, hippocampus, and rostral anterior cingulate cortex (e.g., Johnstone et al., 2006).

No clear evidence exists with respect to ERP markers of specific auditory categories in the literature. The semantic aspects of music and language are indexed by N400 components in a similar way their syntactic structure follows linguistic processing (Minati et al., 2008). Music-syntactically irregular chords elicit an early right anterior negativity (ERAN), while speech-syntactically irregular sentences elicit a left anterior negativity (ELAN). Semantic incongruence would elicit an N400, while harmonic semantic incongruence would elicit a slightly later N500 (Koelsch, 2005). Proverbio et al. (2020a) compared processing of affective vocalizations (e.g., laughter and crying) with music and found that the P2 peak was earlier in response to vocalizations than in response to music, while LP was greater to vocalizations than to music. Source modeling using swLORETA suggested that among N400 generators, the left middle temporal gyrus and the right uncus responded to both music and vocalizations, the right parahippocampal region of the limbic lobe and the right cingulate cortex were active during music listening, and the left superior temporal cortex only responded to human vocalizations. In summary, the available knowledge of the semantic-specific ERP markers of the various types of sounds is not very clear to date.

The present study aimed at assessing reliable category-specific markers, by recording EEG/ERP activity in a large group of human participants. The specific paradigm not involving a motor response toward stimuli of interest but enabling the simultaneous recording of brain signals elicited by a large variety of sensory stimuli in the same male and female subjects seems to be an unprecedented case in the literature. This study may offer potential advancements that can be reached in future BCI research using extremely reliable ERP components.

## Materials and methods

### Participants

A total of 30 healthy right-handed students (15 male and 15 female students) participated in this study as unpaid volunteers. They were recruited via Sona Systems and earned academic credits for their participation. All of them were right-handed, as determined by the Edinburgh Inventory Questionnaire. The experiments were conducted after obtaining written and informed consent from each participant. All the subjects had a normal or corrected-to-normal vision and did not report a history of neurological or psychiatric diseases or drug abuse. They also had normal hearing and *no* self-reported current or past *deficits* in language comprehension, *reading*, or spelling. The experiment was conducted in accordance with international ethical standards and was approved by the Local University Ethics Committee (prot. number RM-2021-432). The data of some participants were excluded after ERP averaging and artifact rejection procedures because of the excessive ocular or motor artifacts affecting the detection of ERP components. The final sample comprised 20 participants, with mean age of 23.9 (*SD* = 3.34) years.

### Stimuli

Pictures (images) and auditory stimuli (sounds) were used as a source of stimulation, which were the same stimuli used in a machine learning study (Leoni et al., 2021), as follows:

- Images of children’s faces (20 males, 20 females)
- Images of adults’ faces (20 males, 20 females)
- Images of animal faces (40 heads of different mammals)
- Images of dressed bodies (20 males, 20 females)
- Images of words (40 written words)
- Images of familiar objects (40 non
- manipulable objects)
- Images of checkerboards (40 different checkerboards)
- Sounds of words (20 male voices, 20 female voices)
- Sounds of emotional vocalizations (40 different voices of crying/laughter/surprise/fear)
- Musical sounds (40 short sequences of piano sounds).

**FIGURE 1:**
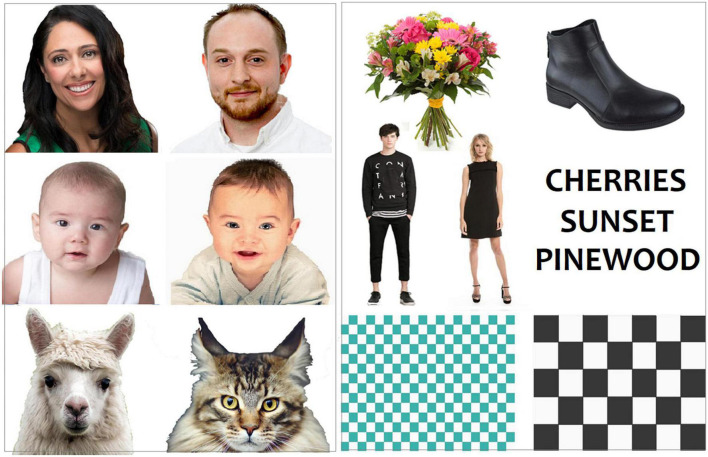
Some examples of visual stimuli belonging to the various categories of living and non-living entities.

The whole picture set comprised 280 images belonging to seven categories (40 images per category). The auditory stimulus set comprised 120 auditory stimuli belonging to three categories (40 sound files per category). The stimuli were matched for size and perceptual familiarity. The stimulus size was 18.5 × 13.5 cm. All the images were in color with a white background and were presented at the center of the screen. Some examples of visual stimuli are shown in Figure 1, while some examples of auditory stimuli can be found here: https://osf.io/yge6n/?view_only=27359c84b8ad449cb4755675c9e221ca. Pictures consisted of people matched for sex and age. All the pictures were equiluminant, as assessed by a repeated measures ANOVA applied to the mean luminance values, recorded using a Minolta CS-100 luminance meter, across categories [*F*(6, 234) = 1.59; *p* = 0.15]. A total of 40 familiar written Italian words were used as linguistic stimuli. Their frequency of use was assessed through an extensive online database of word frequency. The absolute word frequency of use was 176.6, which indicates a fair level of familiarity. Linguistic auditory stimuli comprised 40 words, voiced by two women and two men and recorded through *Huawei P10 smartphone* and *iPhone7* audio recorders. Emotional vocalizations were instead taken from a previously validated database (Proverbio et al., 2020a). The intensity of auditory stimuli ranged between 20 and 30 dB; stimuli were normalized and leveled to avoid variations in intensity or volume by Audacity audio editor software.

### Procedure

The participants comfortably sat inside an anechoic and faradized cabin at 114 cm of distance from an HR VGA color monitor, which was located outside the cabin. The participants were asked to keep their gaze on the fixation point located at the center of the screen and to avoid any ocular or body movements. Visual stimuli were presented in a random order at the center of the screen in eight different runs; auditory stimuli were presented in a random order in four different experimental runs. The stimulus duration was 1,500 ms, while the inter-stimulus interval (ISI) randomly varied 500 ± 100 ms. The experimental procedure was preceded by a training phase. Auditory stimuli were delivered through a pair of headphones (*Sennheiser Electronic GmbH*). The stimuli were followed by an empty gray screen acting as an ISI and meant to cancel possible retinal after-images related to the previous stimulation. An inter-trial interval of 2 s prompted an imagery condition, whose data are discussed elsewhere. During auditory stimulation, the participants gazed at the fixation point and looked at the same background (a light gray screen) on which visual stimuli appeared, and that was left empty during the ISI. To maintain the attention of the participants toward stimulation, they were informed that at the end of the experiment, they would be given a questionnaire on the nature of the stimuli observed.

### Signal acquisition

The EEG was continuously recorded from 126 scalp sites at a sampling rate of 512 Hz and according to the 10/5% system. EEG data were continuously recorded in DC (through *ANT Neuro* amplifiers). Amplifier features were as follows: referential input noise < 1.0 μV rms; referential input signal range = 150–1000 mV pp; input impedance > 1 GOhm; CMRR > 100 dB; maximum sampling rate = 16.384 Hz across all referential channels; resolution = 24 bit; and bandwidth DC (0 Hz)–0.26* sampling frequency. Horizontal and vertical eye movements were also recorded. Averaged ears served as the reference lead. The EEG and electro-oculogram (EOG) were amplified with a half-amplitude bandpass of 0.016–70Hz. Electrode impedance was maintained below 5kΩ. The EEG was recorded and analyzed by EEProbe recording software (ANT software, Enschede, The Netherlands). Stimulus presentation and triggering were performed using by EEvoke software for audiovisual presentation (ANT software, Enschede, The Netherlands). EEG epochs were synchronized with the onset of stimulus presentation.

### Electroencephalogram preprocessing

A computerized artifact rejection criterion was applied to discard epochs in which eye movements, blinks, excessive muscle potentials, or amplifier blocking occurred. The artifact rejection criterion was a peak-to-peak amplitude exceeding 50 μv and the rejection rate of ∼5%. ERPs were averaged offline from -100ms before to 1,500 ms after the stimulus onset. *Averaging* is the most commonly used method for averaging out background noise, that is, for improving the signal-to-noise ratio (SNR) of evoked potentials, which are hidden into the EEG oscillatory waves (Aunon et al., 1981; Regan, 1989). ERP averages were baseline-corrected with reference to the average baseline voltage over the interval of -100 to 0ms. To apply averaging on EEG signals, the full-length record (raw continuous.cnt file) was cut into time-aligned individual single trials synchronized with the stimulus onset and modeled as follows:


X=is+ini

where s_i_ is the ERP signal and n_i_ is the background EEG noise.

If *m* is the number of trials (*m* = 40 in this case) and t is the number of samples of each ERP epoch (768 time points at a sampling rate of 512 Hz, corresponding to 1.5 s), the ERP matrix can be written as follows:


$$A_{\left(m\times t\right)}=\left[\begin{array}{c} x_{1}\\ \begin{array}{c} x_{2}\\ \vdots \end{array}\\ x_{m} \end{array}\right]=\left[\begin{array}{ccc} a_{1,1} & \cdots & a_{1,t}\\ \vdots & \ddots & \vdots \\ a_{m,1} & \cdots & a_{m,t} \end{array}\right]$$

where *x*_*i*_ is defined as a vector of length 768 time points representing the i-th trial. Thus, the resulting ERP-averaged vector is of length t and defined as follows:


$$E\,R\,P_{\left(1\times t\right)}=[\begin{array}{ccc} e_{1} & \cdots & e_{768}] \end{array}\mathrm{where}\mathrm{each}\mathrm{entry}\mathrm{is}ERP_{i}=\frac{1}{m}\sum_{k=1}^{m}a_{\left(i,k\right)}$$

**FIGURE 2:**
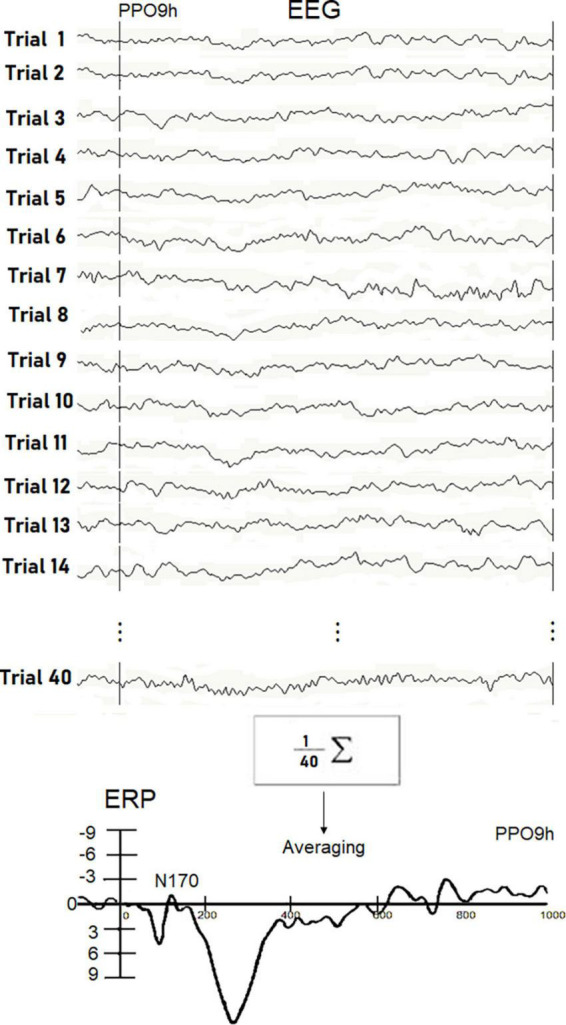
Examples of real EEG trials recorded in response to visually presented words in individual Ss5. The N170 peak reflecting orthographic processing was extracted throughout the averaging procedure since it was hidden in the EEG signals. Unlike P300, the detection of small potentials is not possible through single trial analysis. The waveform recorded in the Ss5 in response to written words at the PPO9h site is displayed at the bottom.

In Figure 2, description of the averaging procedure used for a single experimental subject in a single stimulation condition is displayed. ERP components were identified and measured with reference to the average baseline voltage over the interval of -100 to 0msat the sites and latencies at which they reached their maximum amplitudes.

The electrode selection criteria for ERP measurement was based on the ERP literature (see Tables 1–3 for details about specific time ranges and electrode sites) and on the observed timing and topographic distribution of ERP responses. Precise criteria were as follows: when a component (e.g., visual P2 or P300) showed its maximum amplitude at sites along the midline, two electrodes were selected, along that line, where the electrical potential reached the maximum voltage. When a component (e.g., N2 or AN) showed to be multifocal, with focus both along the midline and laterally over the two hemispheres, multiple electrodes were selected along the midline and on homologous sites of the left and right hemisphere, where the electrical potential reached its maximum amplitude. When a component showed to be focused over the two hemispheres (e.g., N170 or N400), two homologous pairs of left and right electrodes were selected, where the electrical potentials reached their maximum amplitude. The time window of measurement of ERP responses was centered on the peak of maximum amplitude (i.e., the inflection point on the curve), ± a time range depending on the duration of the ERP response. This interval ranged from the 40 ms of highly synchronized N80 and N170 responses (±20 ms from the deflection point) to the 300 ms of the slow components (±150 ms from deflection point, or the plateau midpoint).

ERP data on each channel were entered into the analysis as levels within the factor. ERP waves were filtered offline with a bandpass filter of 0.016/30 Hz for illustration purposes.

### Feature extraction

The mean area amplitude values of the ERP components of interest were subjected to repeated-measure ANOVAs whose factors of variability were stimulus category (depending on the stimuli of interest), electrode (depending on the component of interest), and, where possible, hemisphere (left, right). Tukey (HSD) *post hoc* comparisons (*p* < 0.01; *p* < 0.001) were used for contrasting means. The statistical analyses performed are detailed in the following text.

*Living stimuli* (adult faces, animal faces, and infant faces): The mean area amplitude of the N170 response was recorded from occipitotemporal sites (PPO9h, PPO10h, P7, P8) in the 150–190-ms temporal window. The mean area amplitude of the anterior N2 response was recorded from anterior frontal and centroparietal areas (AFp3h, AFp4h, Fpz, Cpz, Cz) in the 250–350-ms temporal window. The mean area amplitude of the P2 response was recorded from centroparietal sites (Cpz, Pz) in the 300–350-ms temporal window. The mean area amplitude of the P300 response was recorded from frontal midline sites (AFz, Fz) in the 400–600-ms temporal window. The mean area amplitude of centroparietal positivity (CPP) was recorded from centroparietal sites (Cpz, Pz) in the 400–600-ms temporal window. The mean area amplitude of the late CPP (LCPP) response was recorded from the same sites in the 600–900-ms temporal window. The mean area amplitude of the AN was recorded from anterior/frontal areas (AFp3h, AFp4h, AFz, Fpz, Fz) in the 200–600-ms time window. The mean amplitude area of the anterior positivity was recorded from frontocentral sites (AFz, Fz) in the 600–800-ms time window.

*Non-living stimuli* (words, checkerboards, and objects): The mean area amplitude of the N80 response was recorded from mesial occipital sites (Oz, Iz) in the 90–130-ms temporal window. The mean area amplitude of the N170 response was recorded from occipitotemporal sites (PPO9h, PPO10h, P7, P8) in the 150–190-ms temporal window.

*Auditory stimuli* (emotional vocalization, music, and words): The mean area amplitude of the P2 response was recorded from frontocentral and central sites (FFC1h, FFC2h, C1, C2) in the 150–300-ms temporal window. The mean area amplitude of P300 response was recorded from frontocentral and central sites (FFC1h, FFC2h, C1, C2) in the 400–500-ms temporal window. The mean area amplitude of the N400 response was recorded from centroparietal and parietal sites (CCP1h, CCP2h, P3, P4) in the 450–650-ms temporal window. The mean area amplitude of the anterior negativity (AN) response was recorded from frontal sites (AF3, AF4, AF7, AF8) in the 400–600-ms temporal window. The mean area amplitude of late positivity (LP) was recorded from frontal sites (AF3, AF4, AF7, AF8) in the 900–1,200-ms temporal window.

*Auditory vs. visual stimuli*: The mean area amplitude of the PN300 response was recorded from frontal and central sites (Fz, Cz) in the 200–400-ms temporal window in response to auditory and visual stimuli.

## Classification (results)

### Event-related brain potential data

**FIGURE 3:**
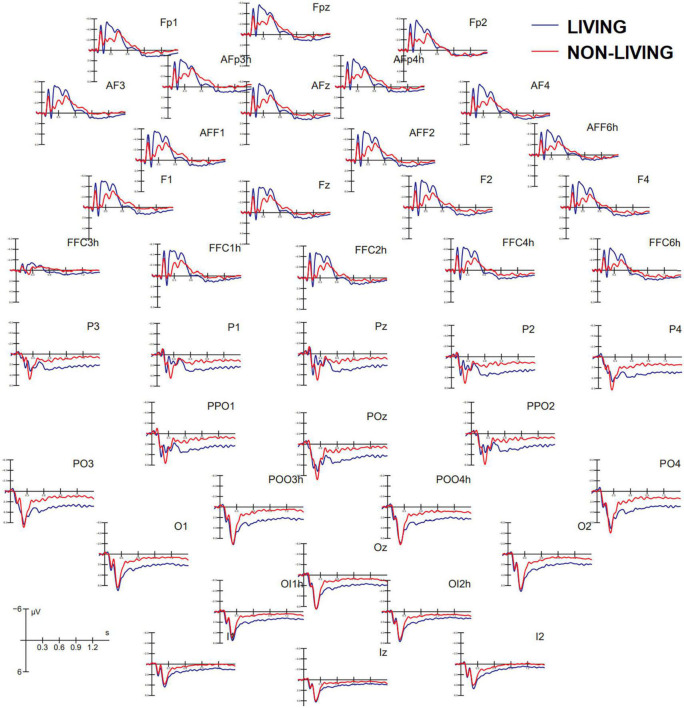
Grand average ERP waveforms recorded at anterior **(top)** and posterior **(bottom)** scalp sites as a function of stimulus category. ERPs to living items were obtained by averaging together ERPs elicited by faces, bodies, and animals, while ERPs to non-living items were obtained by averaging ERPS elicited by checkerboards, written words, and objects.

Figure 3 shows grand average ERP waveforms recorded at anterior and posterior, and left and right sites as a function of the semantic category belonging to visual stimuli (living vs. non-living). A great diversity in the morphology of waveforms can be observed, possibly indicating the biological relevance of faces, bodies, and animals vs. other non-living objects, which was associated with larger amplitudes to the former at both posterior and anterior scalp sites. These differences might be recognized in future by machine learning algorithms (e.g., MATLAB modeling and classification tools) provided with time and scalp site constraints, but further investigation is needed in this regard.

#### Visual stimuli: Living

**FIGURE 4:**
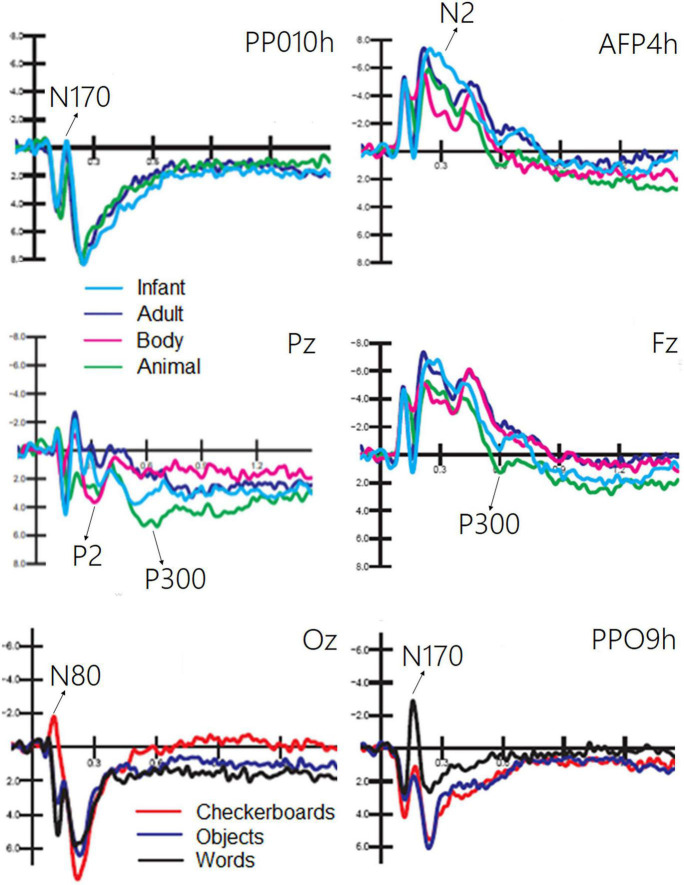
Grand average ERPs recorded at different electrode sites as a function of sensory modality and stimulus category. The ERP components act as reliable markers of semantically distinct perceptual processing, as proved by statistical analyses.

The ANOVA performed on the amplitude of the occipitotemporal N170 (150–190 ms) response showed the significance of category [*F*(2, 38) = 8.54, *p* < 0.001; ε = 1; η^2^*_*p*_* = 0.37], with larger responses to human faces than to animal faces, as shown by *post hoc* comparisons (*p* < 0.001). The ANOVA performed on the anterior N2 response (250–350 ms) showed the significance of category [*F*(3, 87) = 19.58, *p* < 0.001; ε = 1; η^2^*_*p*_* = 0.50] with larger (*p* < 0.002) responses to infant faces than to animal and adult faces. In turn, the N2 response was larger to faces (*p* < 0.0001) than to bodies. The ANOVA performed on the centroparietal P2 response (300–350 ms) revealed the significance of category [*F*(3, 87) = 4.62, *p* < 0.005; ε = 1; η^2^*_*p*_* = 0.26]. *Post hoc* comparisons showed that the P2 response was much larger to bodies (*p* < 0.0001) than to infant, adult, and animal faces. In turn, the P2 response was significantly larger to animal faces than to adult faces. The ANOVA performed on the frontal P300 response (400–600 ms) showed the significance of category [*F*(3, 57) = 11.26, *p* < 0.001; ε = 1; η^2^*_*p*_* = 0.28]. *Post hoc* comparisons showed that the P300 response was larger (*p* < 0.001) to animal faces than to bodies, adult, or infant faces (*p* < 0.05). Moreover, the amplitude recorded in response to infant faces was significantly larger than that to adult faces. These findings are illustrated in Figure 4.

#### Visual stimuli: Non-living

The ANOVA performed on the occipital N80 (90–130 ms) response revealed the significance of category [*F*(2, 38) = 24.70, *p* < 0.0001; ε = 0.72, adjusted *p* = 0.0001; η^2^*_*p*_* = 0.56], as illustrated in Figure 4 (lower part). *Post hoc* comparisons showed that N80 was larger (*p* < 0.001) to checkerboards than to words or objects. The ANOVA performed on the N170 (150–190 ms) response showed the significance of category [*F*(2, 38) = 29.95, *p* < 0.001; ε = 0.86, adjusted *p* = 0.0001; η^2^*_*p*_* = 0.61]. The N170 response was larger (*p* < 0.001) to words than to checkerboards, and larger to checkerboards than to objects. The N170 response was larger over the left than over the right hemisphere [*F*(1, 19) = 7.62, *p* < 0.05; ε = 1; η^2^*_*p*_* = 0.29]. Moreover, the interaction of category × hemisphere [*F*(2, 38) = 62.72, *p* < 0.001; ε = 1; η^2^*_*p*_* = 0.40] and relative *post hoc* comparisons showed that while the N170 response was significantly larger to words than to checkerboards or objects over the left hemisphere, there was no significant effect of category over the right hemisphere. This finding suggests the sensitivity to orthographic properties of the left occipitotemporal area.

#### Visual stimuli: Living (late responses)

**FIGURE 5:**
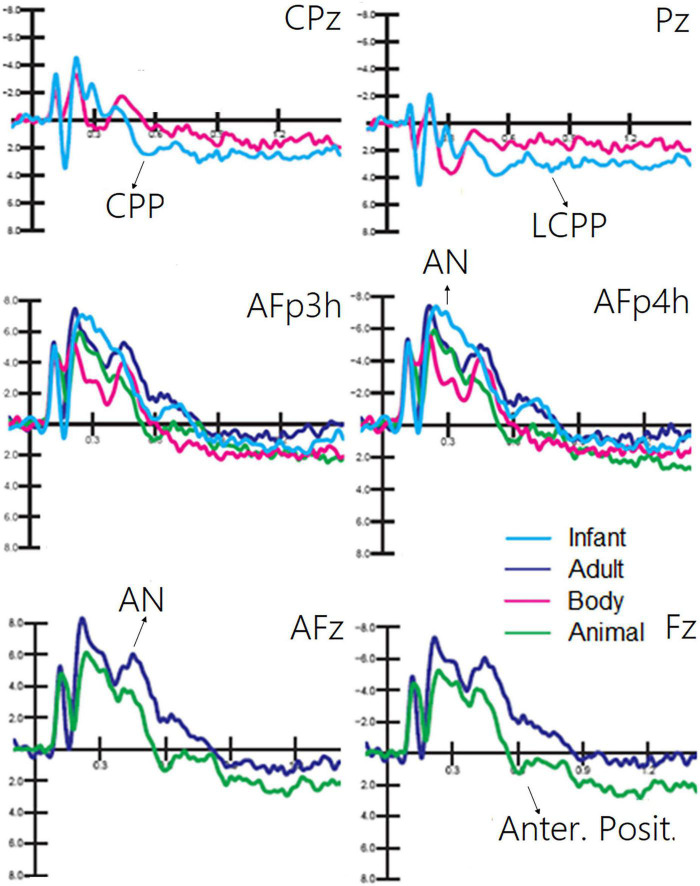
Grand average ERP waveforms showing late latency ERP components sensitive to living visual categories.

The ANOVA performed on CPP (400–600 ms) showed the significance of category [*F*(1, 19) = 9.5, *p* < 0.006; ε = 1; η^2^*_*p*_* = 0.33], with much larger CPP responses to infant faces than to human bodies. The relative ERP waveforms can be observed in Figure 5. The ANOVA performed on the amplitude values of LCPP (600–900 ms) revealed the significance of category [*F*(1, 19) = 35.36, *p* < 0.02; ε = 1; η^2^*_*p*_* = 0.19], with much larger LCPP responses to infant faces than to body stimuli. The ANOVA performed on AN revealed the significance of category [*F*(1, 19) = 21.62, *p* < 0.0001; ε = 1; η^2^*_*p*_* = 0.53]. *Post hoc* comparisons showed that AN to human faces, especially infant faces (*p* < 0.0001), was much larger than that to bodies and animal faces. The ANOVA performed on the amplitude of anterior positivity (AP) revealed a significant effect of category [*F*(1, 19) = 4.5, *p* < 0.001; ε = 1; η^2^*_*p*_* = 0.24], with much larger amplitudes to animal than to adult faces (−0.34 μV, SE = 0.49), as illustrated in Figure 5.

#### Auditory stimuli

**FIGURE 6:**
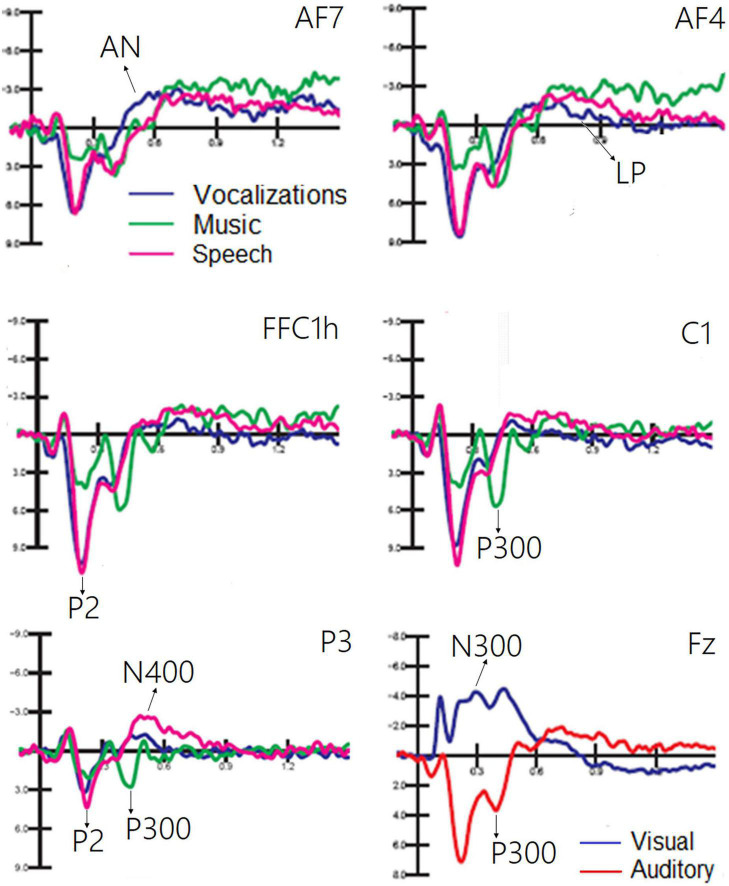
Grand average ERP waveforms relative to the auditory processing of vocalizations, music, and speech. A large PN300 deflection sensitive to stimulus sensory modality (visual vs. auditory) is shown in the **(bottom)** right part of the figure.

**FIGURE 7:**
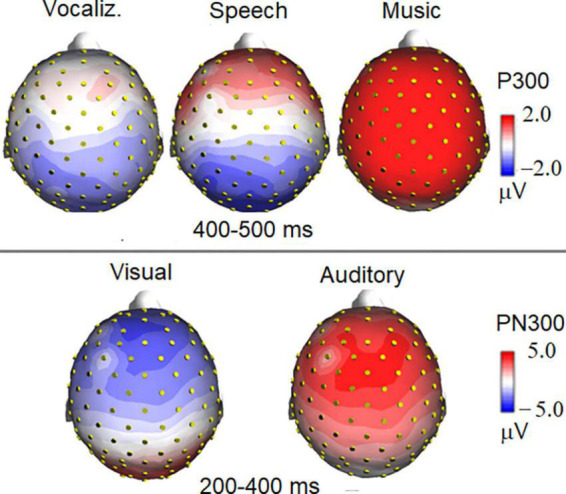
Isocolor topographical maps of surface voltage recorded in the P300 and PN300 latency range. P300 to auditory stimuli was strongly modulated in amplitude by stimulus category **(top)**, being it much larger to musical than to vocal stimuli. The bottom part of the figure illustrates the effect of sensory modality (visual vs. auditory) on the topographical distribution of PN300 wide deflection.

The ANOVA performed on the frontocentral P2 (150–300 ms) response revealed a significant effect of category [*F*(1, 19) = 64.11, *p* < 0.0001; ε = 0.80; adjusted *p* = 0.0001; η^2^*_*p*_* = 0.52], as shown in Figure 6. *Post hoc* comparisons showed that P2 was much larger (*p* < 0.0001) to emotional vocalizations and speech than to music. The ANOVA performed on the frontocentral P300 response (400–500 ms) showed the significance of category [*F*(2,38) = 18.99, *p* < 0.0001; ε = 0.98; adjusted *p* = 0.00001; η^2^*_*p*_* = 0.50], with much larger (*p* < 0.0001) P300 response to music than to vocalizations or words. The topographical maps in Figure 7 (top) clearly show this dramatic difference in P300 amplitude across stimulus categories. The ANOVA performed on centroparietal N400 (450–650 ms) amplitude values showed the significance of category [*F*(2, 38) = 13.44, *p* < 0.0001; ε = 0.94, adjusted *p* = 0.00006; η^2^*_*p*_* = 0.415] with larger N400 responses to speech than (*p* < 0.0001) to music or vocalizations (*p* < 0.01). The ANOVA performed on anterior negativity (AN, 400–600 ms) showed the significance of category [*F*(2, 34) = 11.82, *p* < 0.001) with larger (*p* < 0.0001) AN responses to emotional vocalizations than to music or words. The ANOVA performed on the anterior LP amplitude (900–1200ms) showed the significance of category [*F*(2, 38) = 8.95, *p* < 0.0006; ε = 0.83, adjusted *p* = 0.001; η^2^*_*p*_* = 0.32]; *post hoc* comparisons showed larger (*p* < 0.0001) LP potentials to emotional vocalizations and words than to music. Therefore, this component proved to be strongly sensitive to human voice.

#### Auditory vs. visual stimuli

The ANOVA performed on PN300 amplitude values showed the significance of category [*F*(1, 19) = 45.10, *p* < 0.001; ε = 1; η^2^*_*p*_* = 0.70]. A much greater negativity was recorded in response to pictures than in response to auditory stimuli, as shown in ERP waveforms of Figure 6 (bottom). Furthermore, the interaction of category × electrode [*F*(1, 19) = 11.21, *p* < 0.005; ε = 1; η^2^*_*p*_* = 0.37] indicated an anterior distribution for negativity, and a more posterior distribution for positivity, as can be observed in the topographical maps in Figure 7.

## Discussion

The aim of this study was to identify reliable electrophysiological markers associated with the perception of visual and auditory information of basic semantic categories, recorded in the same participants in order to provide comparative signals recognizable by classification systems (BCI) to be developed for patients or healthy device users. In addition, we aimed to compare the accuracy with which statistical analyses of amplitudes of ERP components (expertly selected) could explain the variance across data due to different categories of stimulation, which enables machine learning systems to automatically categorize the same signals, unconstrained by topography or latency of components.

### Visual perception of living stimuli (adult and infant faces, animals, and bodies)

In agreement with previous studies (e.g., Bentin et al., 1996; Proverbio et al., 2010; Takamiya et al., 2020), a large occipitotemporal N170 component was found in response to adult and infant faces, which was smaller for non-face stimuli. The N170 response was larger to human than to animal faces, as also found by Farzmahdi et al. (2021). However, another ERP study (Rousselet et al., 2004) found that the N170 response to animal and human faces shared a similar amplitude, but the response to human faces was earlier in latency. Visual perception of infant faces elicited a greater anterior N2 response (250–350 ms) than elicited by adult faces, animal faces, and bodies, strongly supporting previous ERP studies (Proverbio et al., 2011b; Proverbio and De Gabriele, 2019; Proverbio et al., 2020b). The N2 response to infant faces was thought to reflect the brain response to baby schema, in particular the activity of reward circuits located within the orbitofrontal cortex, and be sensitive to pedomorphic features of the face (Kringelbach et al., 2008; Glocker et al., 2009; Parsons et al., 2013). Visual perception of human bodies was associated with a greater parietal P2 (300–350 ms) response than visual perception of infant and adult faces, and whether animal faces elicited a larger anterior P300 (400–600 ms) response than bodies and adult faces. Similarly, Wu et al. (2006) found profound P2 and P300 responses in an ERP study involving the perception and imagery of animals. In another ERP study, contrasting visual perception of animals and objects were obtained (Proverbio et al., 2007); they found that the P300 response was larger to animal images than to object pictures. In detail, processing images of animals was associated with faster RTs, larger occipitotemporal N1 components, and larger parietal P3 and LP components (fitting with Kiefer, 2001). Again, evidence was provided for a clear dissociation between the neural processing of animals and that of objects. In addition, infant faces elicited a greater occipitoparietal P2 response (280–380 ms) than animal faces. Again, a centroparietal positivity (CPP, 400–600 ms) showed a greater amplitude in response to infant faces than to bodies, similar to the later LCPP deflection. Data also showed that visual processing of human faces (infant and adult ones) was associated with a larger AN (200–400 ms) than bodies. Finally, animal faces elicited a greater frontal P300 (600–800 ms) than adult faces. Overall, data provided reliable class-specific markers, for instance, N170 for faces, anterior N2 for infants, centroparietal P2 for bodies, and P300 for animals, in unprecedented comparison across ERP signals recorded in the same individuals. A summary of all ERP markers identified in the present study is provided in Table 5.

**TABLE 5:** Functional properties of visual and auditory components of ERPs showing a statistically significant (99.99% or higher) sensitivity to a specific semantic category of stimulation.

Summary					
Category	Peak	Latency (ms)	Scalp area	Electrodes	Functional properties
**Visual perception**					
Human faces	N170	150–190	Occipitotemporal	PPO9h, PPO10h, P7, P8	Larger to human than animal faces (or objects)
Infant faces	N2	250–350	Anterior frontal	AFp3h, AFp4h	Larger to infant than other faces (or bodies)
Bodies	P2	300–350	Centroparietal	Cpz, Pz	Larger to bodies than faces
Animals	P300	400–600	Midline frontal	AFz, Fz	Larger to animal than other faces
Infant faces	CPP	400–600	Centroparietal	Cz, CPz	Larger to infant faces than bodies
Infant faces	LCPP	600–900	Centroparietal	Cz, CPz	Larger to infant faces than bodies
Human faces	AN	200–600	Anterior frontal	AFp3h, AFp4h, AFz, Fpz, Fz	Larger to human than animal faces and bodies
Animals	AP	600–800	Midline frontal	Afz, Fz	Larger to animal than human faces
Checks	N80	90–130	Midline occipital	Oz, Iz	Larger to checks than words or objects
Words	N170	150–190	Left occipitotemp.	PPO9h, PPO10h, P7, P8	Larger to words than checks or objects
**Auditory perception**					
Human voice	P2	150–300	Frontocentral	FFC1h, FFC2h, C1, C2	Larger to vocalizations and speech than music
Music	P300	400–500	Frontocentral	FFC1h, FFC2h, C1, C2	Larger to music than human voice
Speech	N400	450–650	Centroparietal	CCP1h, CCP2h, P3, P4	Larger to speech than music and vocalizations
Vocalizations	AN	400–600	Anterior frontal	AF3, AF4, AF7, AF8	Larger to vocalizations than speech and music
Human voice	LP	900–1200	Anterior frontal	AF3, AF4, AF7, AF8	Larger to vocalizations and speech than music
Sensory modality	PN300	200–400	Midline fronto/central	Fz, Cz	Negative for visual, positive for auditory

The summary also provides indication about the peaks’ name, latency (in ms), scalp area, and electrode site of recording.

### Visual perception of non-living stimuli (checkerboards, words, and objects)

The results showed that checkerboards elicited larger N80 sensory responses than words and familiar objects. It is largely known that N80, also named C1 response of visual evoked potentials (VEPs), is primarily sensitive to stimulus spatial frequency and check size (Jeffreys and Axford, 1972; Regan, 1989; Bodis-Wollner et al., 1992; Shawkat and Kriss, 1998; Proverbio et al., 2021). We are not aware of any studies in the literature that directly compared the N80 response with spatial frequencies vs. objects or words. In this study, words elicited a larger occipitotemporal N170 response (150–190 ms) than checkerboards and familiar objects. The N170 response was left-lateralized, consistent with a large study showing the orthographic properties of this component, possibly reflecting the activity of the VWFA, located in the left fusiform gyrus (Salmelin et al., 1996; Proverbio et al., 2008; Yoncheva et al., 2015; Canário et al., 2020).

### Auditory perception

The results showed that emotional vocalization and words elicited a greater central P2 response (150–300 ms) than music, which therefore might represent a strong marker for human voice perception. According to the ERP literature, P2 is the earliest response being sensitive to the emotional content of linguistic stimuli and vocalizations (Paulmann and Kotz, 2008; Schirmer et al., 2013), with positive words typically eliciting larger P200 response than neutral words (e.g., Paulmann et al., 2013; Proverbio et al., 2020a). Music elicited a greater frontocentral P300 response (400–500 ms) than emotional vocalizations, while words elicited a larger centroparietal N400 amplitude than music. These findings is consistent with the previous literature reporting larger P300 amplitudes over frontal areas in response to pleasant vs. unpleasant music (Kayashima et al., 2017).

As for the N400 component, which is larger for speech than for non-linguistic auditory stimuli, it seems to share some properties with the centroparietal N400, which reflects semantic and word processing (Kutas and Federmeier, 2009). The left hemisphere asymmetry found in this study is consistent with a large neuroimaging and electromagnetic literature (see Richlan, 2020), as well as clinical data, predicting left lateralization of speech processing. However, left lateralization is not typical of N400 response elicited by semantic incongruence. It should be mentioned that in this case, words were presented individually, without a previous context; therefore, it is conceivable that the N400 response to words reflects speech recognition, rather than violation of semantic expectation. In addition to the previous data, a greater AN (400–600 ms) was recorded in response to emotional vocalizations than to music and words, and a larger LP (900–1,200 ms) that was of greater amplitude during processing of the human voice (i.e., emotional vocalization and words) than during processing of music (see also Proverbio et al., 2020a). ERPs also showed clear markers reflecting stimulus sensory modality: as a whole, visual stimuli elicited a much greater negativity over frontal and central sites (200–400 ms) than auditory stimuli.

### Possible data usage in brain computer interface applications

**FIGURE 8:**
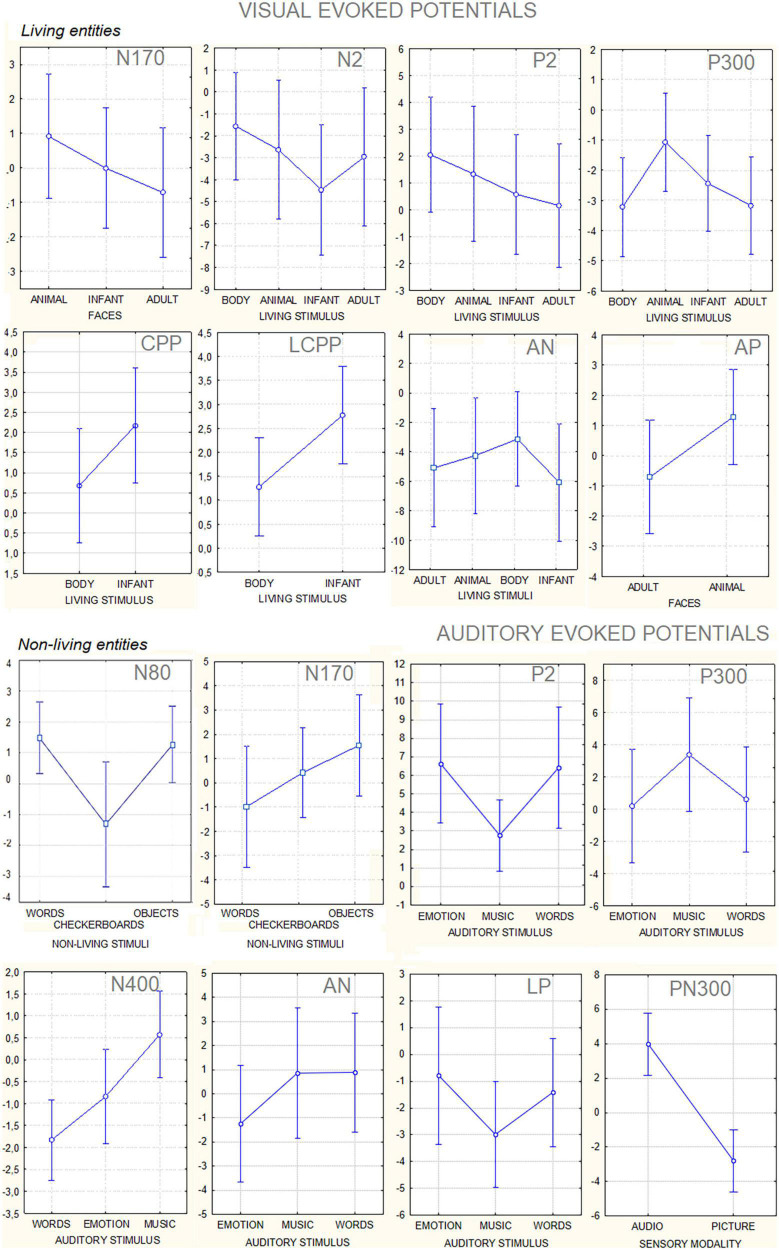
Mean amplitude values (along with standard deviations) in microvolts of the different component ERPS recorded to visual and auditory stimuli. The data are relative to the grand average computed on the whole group of participants.

**FIGURE 9:**
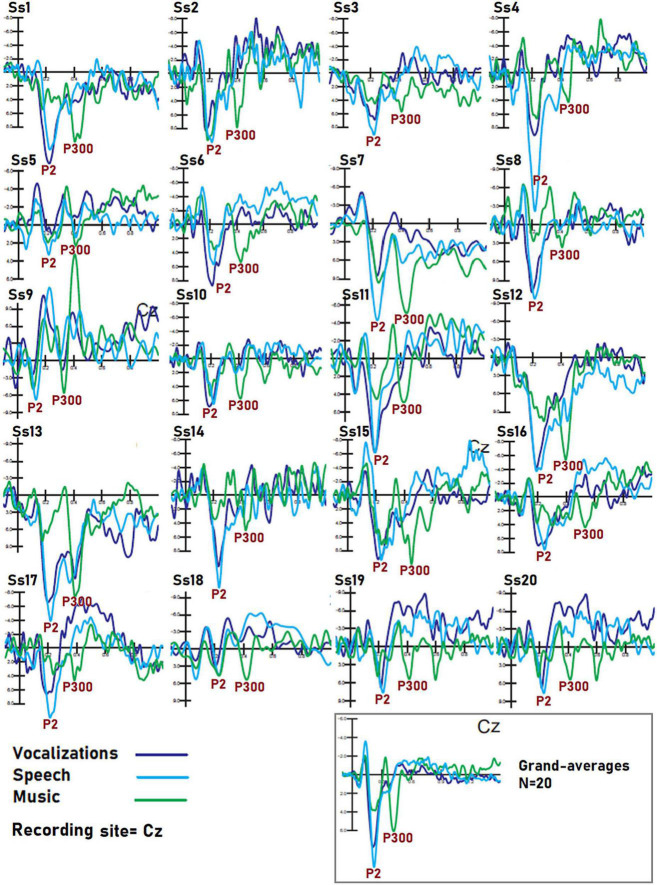
Individual data of subjects. Dataset recorded from 20 participants in response to auditory stimuli of the three classes. The example shows that despite the obvious inter-individual differences in electrical potentials, markers of speech, music, and vocalization processing were visible in each individual, so it is reasonable to assume that BCI performance on each person’s dataset would be still good. Further research is needed to reach a definitive conclusion on this topic.

This paradigm is unique in the literature because it contrasted brain signals to a large variety of perceptual categories in the same participants. To our knowledge, previous comparative data (confronting, e.g., speech, music and voice, or, checkerboards, words, and objects, at the same time) are not available in the ERP literature. For this reason, some of the markers described here are unprecedented, namely, CPP, LCPP, AN, and AP for living categories, PN300 for sensory modalities, and the markers of auditory perception. The ERP markers of category-specific processing here reported were identified through the statistical methods (repeated measures analysis of variance, ANOVA), aided by a neuroscience-based supervised expert analysis. The findings can be hopefully helpful for setting future constraints for unsupervised/supervised machine learning and automated classification systems (e.g., Jebari, 2013; Ash and Benson, 2018; Yan et al., 2021). Figure 8 shows data variance for each statistical contrast among stimulus categories. As can be observed, notwithstanding the obvious inter-individual differences, standard deviation values were strictly homogeneous across stimulation and sensory conditions. This is important for BCI applications that often show some variation among individuals (see, e.g., the waveforms in Figure 9) to show that mixing data from multiple individuals does not reduce the distinguishability of category-specific ERP signals. The ERP components identified in this study might be tested with the same algorithms of P300 speller, which is a visual ERP-based BCI system, which can elicit P300 ERP components *via* an oddball paradigm. It should be considered, however, that statistical significances from ANOVA indicate that there was a statistically significant difference between mean potentials, but it still does not imply any “accuracy” unless the component would be used for BCI classification. This accuracy should be tested by further BCI studies whose architecture could be designed in light of the present findings.

Overall, the categorization based on statistical analyses and expert supervision seems superior to the machine learning system applied to the same stimuli (Leoni et al., 2021), especially for discriminating living stimuli (i.e., faces of various age, animals, and bodies), but does not possess the automaticity and replicability of the latter. Above all, it requires strong human supervision for site and latency selection, based on consolidated knowledge. However, while highly discriminative ERP components may be useful for feature extraction or the selection stage in algorithm design or for psychophysiological explanations of brain activities, direct comparison between these two methods does not seem appropriate at present since empirical BCI applications of the components just described have not yet been developed. We hope that the knowledge provided by using this methodology may provide space–time constrains for optimizing future artificial intelligence (AI) systems devoted to reconstructing mental representations related to different categories of visual and auditory stimuli in an entirely automatic manner (Power et al., 2010; Jin et al., 2012; Lee et al., 2019).

## Data availability statement

The original contributions presented in this study are included in the article/supplementary material, further inquiries can be directed to the corresponding author.

## Ethics statement

The studies involving human participants were reviewed and approved by the Ethics Committee of University of Milano-Bicocca (CRIP, prot. number RM-2021-432). The patients/participants provided their written informed consent to participate in this study. Written informed consent was obtained from the individual(s) for the publication of any identifiable images or data included in this article.

## Author contributions

AP conceived and designed the study, led the manuscript writing, supervised the data recording and analysis, and interpreted the data. MT collected the data, performed the statistical analysis, and plotted the figures. KJ contributed to the original conception, prepared the experimental stimuli, and paradigm. All authors contributed to the writing and interpretation.
